# Structural characteristics of neutral polysaccharides purified from coix seed and its anti‐insulin resistance effects on HepG2 cells

**DOI:** 10.1002/fsn3.4402

**Published:** 2024-09-08

**Authors:** Guozhen Wu, Shuang Liu, Zhenqiang Wang, Xiao Wang

**Affiliations:** ^1^ School of Pharmaceutical Sciences Shandong University of Traditional Chinese Medicine Jinan P.R. China; ^2^ Key Laboratory for Applied Technology of Sophisticated Analytical Instruments of Shandong Province, Shandong Analysis and Test Center Qilu University of Technology (Shandong Academy of Sciences) Jinan P.R. China; ^3^ Key Laboratory for Natural Active Pharmaceutical Constituents Research in Universities of Shandong Province, School of Pharmaceutical Sciences Qilu University of Technology (Shandong Academy of Sciences) Jinan P.R. China

**Keywords:** anti‐insulin resistance effect, coix seed, PI3K/AKT signaling pathway, polysaccharides, structural characteristics

## Abstract

Coix seed is recognized as a functional medicinal food due to its valuable biological activities, with polysaccharides being the primary active compounds. In this study, an ultrasonic‐assisted enzymatic extraction technique was employed, and response surface methodology was used to optimize the yield of polysaccharides to 9.55 ± 0.26%. A novel neutral polysaccharide, CSPsN‐1, was purified with a molecular weight of 7.75 kDa. CSPsN‐1 was composed of arabinose, galactose, glucose, xylose, and mannose in molar ratios of 0.48: 7.92: 86.39: 2.42: 2.79. Its backbone composed of →4)‐α‐D‐Glc*p*‐(1→ and →3,4)‐α‐D‐Glc*p*‐(1→ units, with terminal residues of α‐D‐Glc*p*. In vitro experiments, CSPsN‐1 enhanced glucose consumption in insulin‐resistant HepG2 cells and upregulated GLUT4 expression by activating the PI3K/AKT signaling pathway. These findings suggest that CSPsN‐1 holds significant promise as a functional ingredient for treating insulin resistance and related metabolic disorders.

## Graphical abstract

In this study, CSPsN‐1, a polysaccharide with a molecular weight of 7.75 kDa, was extracted from coix seed using an ultrasonic‐assisted enzymatic method. Its structure comprises →4)‐α‐D‐Glc*p*‐(1→ and →3,4)‐α‐D‐Glc*p*‐(1→ units, with terminal α‐D‐Glc*p* residues. CSPsN‐1 enhances glucose uptake by activating the PI3K/AKT/GLUT4 pathway, suggesting its potential as a functional ingredient for treatment of insulin resistance and related metabolic disorders.

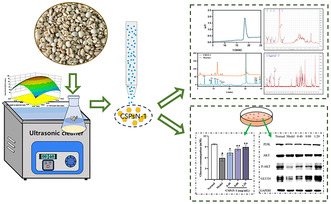

## 1 INTRODUCTION

Coix seed, derived from the mature seeds of *Coix lacryma‐jobi* L., is widely cultivated in warm regions of Asia, Africa, and the Mediterranean coast. In several Asian regions, coix seed is valued not only as a diary staple but also as a key ingredient in fermentation and brewing processes (F. Zhu, 2017). Compared with other cereals, coix seed has a rich history in traditional medicine, where it is commonly used to enhance spleen and stomach functions, promote urinary excretion, and provide anti‐inflammatory and antispasmodic effects (Liu et al., 2019). Furthermore, research has demonstrated that coix seed has a significantly lower glycemic index (GI) and glycemic load than other grains, such as brown rice, making it a valuable resource for regulating hyperglycemia (Lin et al., 2010). These properties underscore the importance of coix seed as a functional food with potential therapeutic applications in both nutrition and medicine.

Polysaccharides, a diverse class of macromolecules found in plants, animals, algae, and fungi, play critical roles in biomedical functions and nutritional health. They are valuable as low‐calorie foods and dietary fiber, significantly contributing to overall wellness (Wang, Dai, et al., 2022). Numerous studies have demonstrated that polysaccharides can modulate insulin resistance and improve insulin sensitivity, particularly through their blood sugar‐lowering effects (Tang et al., 2023). In coix seed, polysaccharides are the principal active components, exhibiting a range of functional properties, including anti‐inflammatory, analgesic (Sui & Xu, 2022), and hypoglycemic activities (Chen et al., 2019). Previous studies have explored the hypoglycemic activity of coix seed polysaccharides; however, the precise structural characteristics responsible for these effects remain poorly defined (Xia et al., 2021). To better utilize coix seed polysaccharide for improving blood sugar regulation, it is essential to study and clarify its structure characteristics.

Efficient extraction techniques for coix seed polysaccharides are key to preserving their structural integrity and bioactivity, which are crucial for subsequent structural characterization and comprehensive application. Hot water extraction, although common, is hindered by slow processing, low yields, and reagent loss, making it inadequate for modern production needs (Xia et al., 2021; Yin et al., 2020). As a result, alternative methods such as microwave‐assisted extraction, acid–base extraction, ultrasonic‐assisted extraction, and enzymatic extraction have been developed to enhance efficiency (Gao et al., 2022). Among these methods, ultrasonic extraction is particularly effective and practical, as it preserves the structural integrity of polysaccharides, thereby ensuring their functional properties (Chu et al., 2024). Enzymatic extraction, which uses biocatalysts, accelerates the release of polysaccharides from cells (Olawuyi et al., 2020). Combining ultrasonic‐assisted extraction with enzymatic extraction offers enhanced efficiency and versatility, potentially improving the extraction yield of coix seed polysaccharides for broader applications.

Type 2 diabetes mellitus (T2DM) is a complex chronic metabolic disease that significantly threatens modern health (Cole & Florez, 2020). Insulin resistance, a prominent pathogenic mechanism in T2DM, results from abnormalities in insulin signaling pathways at various levels, including the insulin receptor, insulin receptor substrate phosphorylation, and the postreceptor signaling pathways, leading to inadequate insulin responsiveness (Eddouks et al., 2014). Studies have shown that high‐calorie, high‐fat diets are major exogenous factors contributing to insulin resistance, with free fatty acid metabolic derivatives playing a crucial role (Klein et al., 2022). Oral biguanides and sulfonylureas are commonly prescribed for T2DM, but they have adverse effects and their long‐term use increases the financial and physical burden on patients. Recent researches indicate that natural polysaccharides can address these issues and considerably improve treatment for T2DM by enhancing insulin sensitivity and reducing blood glucose levels (Chen, Li, et al., 2023; Tang et al., 2023).

Based on above backgrounds, the study aims to enhance the efficiency of polysaccharide extraction from coix seed by optimizing ultrasonic‐assisted enzymatic extraction conditions. Subsequently, a neutral polysaccharide (CSPsN‐1) was isolated and purified, and its structural properties were characterized. The effects of CSPsN‐1 on insulin resistance in HepG2 cells and its impact on relevant signaling pathways were then evaluated. This work provides valuable insights and guidance for the development of coix seed polysaccharides as health‐promoting foods and potential therapeutic agents.

## 2 MATERIALS AND METHODS

### 2.1 Materials and reagents

Coix seed was purchased from Yaoshengtang Traditional Chinese Medicine Technology Co., Ltd (Changsha, China), and stored at room temperature under dry conditions. Monosaccharide standards (glucose, mannose, galactose, arabinose, and xylose) were bought from Sigma‐Aldrich Co. (St. Louis, MO, USA). Cellulase (400 U/mg), papain (800 U/mg), DEAE‐52 cellulose, and Sephadex G‐50 were obtained from Yuanye Biotechnology (Shanghai, China). Palmitic acid (PA) was manufactured by Kunchuang Technology Development Co., Ltd (Xian, China). DMEM medium, penicillin–streptomycin solution, and fetal bovine serum were purchased from Gibco BRL (Carlsbad, CA, USA). The glucose test kit was purchased from Jiancheng Bioengineering Institute (Nanjing, China). Unless otherwise specified, all remaining reagents were of analytical grade.

### 2.2 Extraction and purification of coix seed polysaccharides

To prepare the experimental sample, dried coix seed was smashed and sieved. Based on preliminary experiments, extraction parameters including ultrasound time, liquid–solid ratio, and complex enzyme amount were selected for single‐factor experiments. Optimization was then achieved using response surface methodology (RSM) with a Box–Behnken design.

The procedure for the ultrasonic‐assisted enzymatic extraction of CSPs was adapted from Wang et al. (2024). Dried coix seed samples were placed into conical flask and immersed in 20 mL of deionized water. The liquid‐to‐material ratio was adjusted to 30:1, 20:1, and 10:1 mL/g by varying the quantity of coix seed. Complex enzymes were added at concentrations of 5%, 6%, and 7%. The mixture underwent ultrasonic extraction (3500 W) at 60°C for 100, 120, and 140 min. After extraction, the solution was filtered and concentrated, followed by precipitation with 80% ethanol. The resulting mixture was centrifuged (8000 rpm, 5 min) and freeze‐dried to obtain crude polysaccharides. The amount of crude polysaccharide was determined using the phenol‐sulfuric acid method (Masuko et al., 2005). The extraction yield (%) was calculated as (W_cp_/W_s_) × 100%, where W_cp_ (g) is the weight of crude polysaccharides extracted, and W_s_ (g) is the weight of the dried coix seed sample.

The crude polysaccharides were extracted using the optimized ultrasound‐assisted enzyme extraction conditions. The crude polysaccharide solution was then redissolved and treated with petroleum ether to remove lipophilic components (Fan et al., 2015). Next, Sevag reagent (chloroform/*n*‐butanol, 4:1, v/v) was repeatedly used to remove free proteins (Long et al., 2020). The coix seed polysaccharides (CSPs) were obtained by freeze drying. For further purification, 5.0 g of CSPs was separated using a DEAE‐52 column. The mobile phase consisted of sodium chloride (NaCl) solutions (0, 0.3, 0.6, and 0.9 M) with a flow rate of 1.0 mL/min. After collection, the fractions were condensed and dialyzed (3500 Da), resulting in two polysaccharides: CSPsN and CSPsA. CSPsN was further purified using a Sephadex G‐50 column to obtain CSPsN‐1. In addition, the protein content in CSPsN‐1 was determined by the Bradford method using BSA as the standard (Bradford, 1976), and in order to confirm the absence of starch, the iodine reagent (0.5% iodine in 5% potassium iodide) test was conducted (Cheong et al., 2016).

### 2.3 Molecular weight (*M*w) determination of CSPsN‐1

The *M*w of CSPsN‐1 was determined utilizing high‐performance gel permeation chromatography (HPGPC) on an Agilent Infinity1260 System (Agilent Technologies, CA, USA), which was equipped with two PL aquagel‐OH columns (7.5 ×300mm) in series. A 50 μL aliquot of the filtered sample solution was injected into the HPGPC column, maintained at a constant temperature of 30°C. Elution was performed with a 0.1 M NaNO_3_ solution at a flow rate of 1 mL/min. Detection was carried out using a refractive index detector (Zhang et al., 2019).

### 2.4 Monosaccharide composition analysis of CSPsN‐1

The monosaccharide composition analysis of CSPsN‐1 was conducted using the ICS 5000+ System from Thermo Fisher Scientific, equipped with a Dionex™ CarboPac™ PA20 column (3.3 mm × 150 mm) and a pulsed amperometric detector. A 10 mg sample of CSPsN‐1 was hydrolyzed with 2 M trifluoracetic acid (TFA) and subsequently dried under nitrogen steam. The hydrolyzed sample was then dissolved in deionized water and filtered through a 0.22 μm membrane. The analysis was performed with an injection volume of 5 μL, a flow rate of 0.5 mL/min, and the column temperature maintained at 30°C (Peng et al., 2024).

### 2.5 Fourier transform infrared (FT‐IR) spectroscopy

The functional groups present in CSPsN‐1 were characterized through FT‐IR spectroscopy with the potassium bromide (KBr) disk method, employing a Nicolet 710 spectrometer (MA, USA). CSPsN‐1 was blended with KBr to form a powder mixture, which was then compressed into 1.0 mm tablets using a vacuum tablet press. The infrared spectra were recorded over the frequency range of 4000 to 400 cm^−1^ (Wang et al., 2014).

### 2.6 Congo Red test

The Congo Red experiment was conducted based on a modified protocol from Zhu et al. (2021). The CSPsN‐1 solution (2.0 mg/mL) was mixed with NaOH solutions of several concentrations (0, 0.1, 0.2, 0.3, 0.4, 0.6, and 0.8 mol/L). After complete mixing and a 1‐hour incubation period, the maximum absorption wavelength of the reaction mixture was measured using a UV–visible spectrophotometer over a range of 200–600 nm. The control sample consisted of the Congo Red solution at a concentration of 200 μmol/L without polysaccharides. A curve was then constructed to depict the change in polysaccharide absorbance, with the maximum absorbance as the dependent variable and the NaOH concentration as the independent variable.

### 2.7 Methylation and gas chromatography–mass spectrometry (GC–MS) analysis

The types and radios of sugar residues in CSPsN‐1 were determined using the Hakomori method (Hakomori, 1964). Briefly, 3 mg samples of CSPsN‐1 was methylated with sodium hydroxide and iodomethane in 1 mL of DMSO. The methylated products were then hydrolyzed with TFA, reduced with NaBH_4_, and acetylated with acetic anhydride. These derivatives were extracted with dichloromethane, and the partially methylated alditol acetates were analyzed by GC–MS. The GC–MS system used was a Thermo Scientific 1300–7000, equipped with an electron ionization source and an HP‐INNOWAX column (30 m × 0.32 mm × 0.25 μm). The initial temperature was set at 140°C for 2 min, then increased to 230°C at a rate of 3 °C/min, and held for 3 min.

### 2.8 Nuclear magnetic resonance (NMR) spectroscopy

For NMR analysis, 35 mg of CSPsN‐1 was dissolved in 0.55 mL of D_2_O (99.9%) in an NMR tube. The 1D NMR (^1^H NMR and ^13^C NMR) and 2D (^1^H‐^1^H COSY and HSQC) spectra of CSPsN‐1 were recorded using an ADVANCE DPX 400 NMR spectrometer (Bruker, Rheinstetten, Germany) (Geng et al., 2024).

### 2.9 Evaluation of improving insulin resistance

#### 2.9.1 Cell culture

HepG2 cells were cultivated in DMEM media supplemented with 10% fetal bovine serum (FBS), 100 U/mL penicillin, and 100 μg/mL streptomycin, and incubated at 37°C with 5% carbon dioxide. When the cells reached approximately 80% confluency, the medium was removed, and the cells were washed twice with 1× PBS. Subsequently, trypsin was added to facilitate cell detachment, and the cells were subcultured at a 1:2 ratio.

#### 2.9.2 Effect of CSPsN‐1 on cell viability and glucose consumption in HepG2 cells

The insulin‐resistant HepG2 cell model was established using previously reported methods with slight modifications (Huang et al., 2019). Initially, the cells were seeded in a 96‐well plate (2 × 10^4^ cells/well) and incubated in a carbon dioxide incubator at 37°C for 24 h. After that, the medium was replaced with DMEM supplemented with 0.6 mM palmitic acid (PA), and the cells were further incubated for an additional 24 h. At last, CSPsN‐1 (0.40, 0.80, and 1.20 mg/mL) dissolved in DMEM containing 0.6 mM PA was added to the 96‐well plates and incubated for another 24 h. Cell viability was assessed using the MTT assay (Yu et al., 2022). Glucose consumption was determined using the glucose oxidase method, following the instructions of the kit manufacturer.

#### 2.9.3 Western blot assays

Total proteins were extracted from HepG2 cells using RIPA buffer supplemented with 10 mM phenyl methane sulfonyl fluoride (PMSF) and phosphatase inhibitor (PI). Following lysis on ice, the protein supernatant extracts were collected by centrifugation (12,000 rpm/min, 10 min), and the protein concentration was determined. The denatured protein samples were then loaded onto a 10% sodium dodecyl sulfated–polyacrylamide gel (SDS‐PAGE) for electrophoresis and subsequently transferred to the PVDF membrane. After blocking the membranes for 1 h with a solution comprising 5% skim milk and 0.1% Tween‐20 tris‐buffered saline, they were incubated overnight at 4°C with primary antibodies, including phosphatidylinositol 3‐kinase (PI3K), protein kinase B (AKT), phosphorylated protein kinase B at serine 473 (P‐AKT, ser 473), glucose transporter type 4 (GLUT4), and glyceraldehyde 3‐phosphate dehydrogenase (GAPDH). Following this, the membranes were further incubated with the corresponding HRP‐conjugated secondary antibody for 1 h. At last, excess antibody was removed, and the expression of protein bands was visualized by chemiluminescence.

### 2.10 Statistical analysis

All data were expressed as the mean ± standard deviation (SD) for the three independent experiments. Statistical analysis and experimental design were performed using Design‐Expert (V8.0.6, Statease Inc., USA). The student's *t*‐test and one‐way ANOVA were employed to analyze statistical differences, with a *p*‐value of less than .05 considered statistically significant.

## 3 RESULTS AND DISCUSSION

### 3.1 Optimization of the extraction conditions for the polysaccharides yield

#### 3.1.1 Influence of different factors on ultrasonic‐assisted enzymatic extraction

Various ultrasonic times (60, 80, 100, 120, and 140 min), liquid‐to‐material ratios (10:1, 20:1, 30:1, 40:1, and 50:1 mL/g), and complex enzyme concentrations (4%, 5%, 6%, 7%, and 8%) were tested to determine their impact on the extraction yield of crude polysaccharides.

As shown in Figure S1a, the extraction yield increased with ultrasonic time, reaching a peak at 120 min. Compared to conventional hot water extraction methods, ultrasonic extraction significantly reduced the required extraction time (Yin et al., 2020). However, extending the ultrasonic extraction time beyond 120 min resulted in a gradual decrease in the yield of crude polysaccharides. This decrease may be attributed to the prolonged exposure leading to polysaccharide degradation and the release of other macromolecular components from the coix seed, which could hinder the diffusion of polysaccharides (Tian et al., 2017).

The liquid‐to‐material ratio is a crucial factor influencing the yield of the extraction process. As the ratio increases, the yield of crude polysaccharides also increases, reaching an optimal point at a ratio of 20:1 mL/g. Beyond this ratio, the yield plateaus and shows minimal change. An appropriate liquid‐to‐material ratio reduces the density and viscosity of the extraction solvent, facilitating the dissolution of more polysaccharide molecules in water. However, further increasing the ratio beyond the optimal point decreases the density of the extraction mixture, resulting in a reduction in the extraction yield (Chen et al., 2015).

Empirical investigations revealed that papain hydrolyzes the free proteins in coix seed, loosening the plant structure, reducing the binding force with raw materials, and facilitating the leaching of polysaccharides (Zhao, Wang, et al., 2016). Simultaneously, cellulase hydrolyzes the cell wall, releasing more polysaccharides (Lin et al., 2016). As shown in Figure S1c, the maximum extraction yield was achieved with a 6% complex enzyme concentration, which was significantly higher than the yields obtained with other enzyme concentrations. Beyond 6%, increasing the enzyme concentration resulted in a decrease in yield. Therefore, a 6% complex enzyme concentration was selected for subsequent experiments.

#### 3.1.2 Fits for the response surface model

The RSM approach was employed to identify the significant factors influencing extraction yield, specifically ultrasonic time (*A*), liquid‐to‐material ratio (*B*), and complex enzyme amount (*C*), based on the single‐factor trials. The ranges for these factors were 100 to 140 min for ultrasonic time, 10:1 to 30:1 mL/g for the liquid‐to‐material ratio, and 5% to 7% for the enzyme concentration. A total of 17 randomized experiments were carried out. The experimental data were subjected to multiple regression analysis, fitting each independent variable and its corresponding response into a second‐order polynomial equation: *Y* (extraction yield of polysaccharides, %) = 94.95 + 5.75 * *A* + 7.68 * *B* + 4.18 * *C* + 4.14 * *A* * *B* – 2.47 * *A* * *C* + 2.10 * *B* * *C* – 11.57 * *A* ^2^–30.33 * *B* ^2^–10.39 * *C* ^2^.

Table S1 presents the experimental results for each dependent variable, demonstrating strong agreement with the second‐order polynomial model. The adequacy of the fit was assessed using the *R* ^2^ value, which was 0.9828, indicating a high degree of correlation between the observed and predicted values. The lack‐of‐fit test was insignificant (*p* > .05), suggesting that the model is adequate at the 95% confidence level. Therefore, all the aforementioned parameters were included in optimizing the extraction of crude polysaccharides from coix seed.

The adaptability and applicability of the response surface model were assessed through an analysis of variation (ANOVA), which confirmed the statistical significance of individual factors and their interactions. The response variable in ANOVA (Table S2) indicated statistical significance, as evidenced by a high *F*‐value and low *p*‐value. The highly significant linear coefficients (*A*, *B*, *C*), interaction coefficients (*AB*), and quadratic coefficients (*A* ^2^, *B* ^2^, *C* ^2^) further highlighted the considerable influence of the independent variables on the production of crude polysaccharides (*p* < .05). The coefficient of variation (CV) value of 5.37% further demonstrated the reliability and precision of the experimental data. These results indicate that the model effectively represents the relationship between the response value (yield of polysaccharides) and the independent variables (ultrasonic time, liquid‐to‐material ratio, and complex enzyme amount).

**FIGURE 1:**
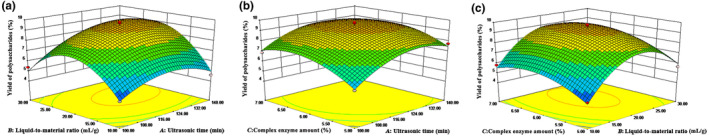
Response surface plots of interactive effects on yield of coix seed crude polysaccharides.

The 3D response surface diagram, as illustrated in Figure 1a–c, was employed to determine the optimal conditions for polysaccharide extraction. The slope of each curve indicates the degree of interaction between the two variables. In Figure 1a, the steep slopes for variables *A* and *B* suggest a strong interaction between these two factors. For the RSM model used to extract crude polysaccharides from coix seed, the optimal parameters were determined to be a complex enzyme concentration of 6.21%, a liquid–material ratio of 20:1 mL/g, and an ultrasonic extraction time of 126 min. Implementing these conditions in three repeated trials resulted in an extraction yield of 9.55 ± 0.26%. These experimental values closely matched the predicted results from the RSM model, suggesting that the RSM model possesses significant predictive capability. This correspondence also confirms that the experimental design procedures are suitable for extracting crude polysaccharides from coix seeds.

### 3.2 Preparation of CSPsN‐1

The CSPs were collected using the ultrasonic‐assisted enzymatic extraction technique under optimal conditions, with subsequent removal of protein and lipid components. The separation and purification of CSPs were performed using column chromatography. Anion exchange column chromatography and Sephadex gel column chromatography are the primary techniques for purifying polysaccharides, allowing thorough separation and purification based on distinct retention times associated with different charges and molecular weights (Peng et al., 2022; Wang, Han, et al., 2022).

**FIGURE 2:**
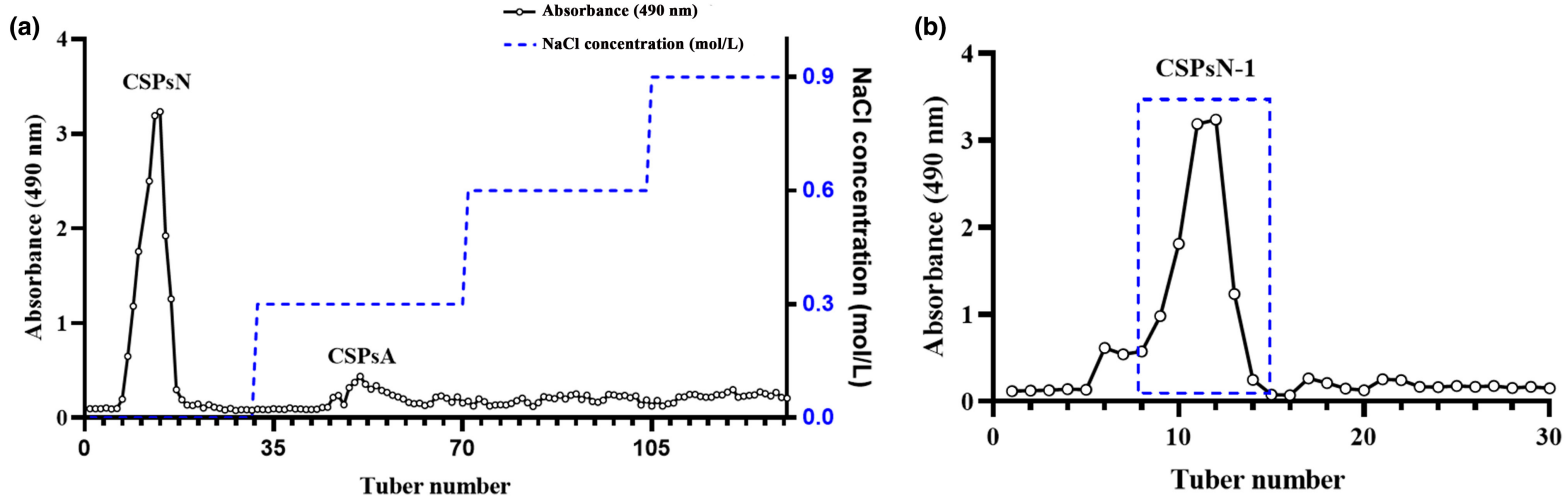
(a) Elution curves of CSPs on a DEAE‐52 column. (b) Elution curves of CSPsN‐1 on a Sephadex G‐50 column.

The initial separation of CSPs was achieved by anion exchange column chromatography, resulting in the detection of two distinct sugar peaks (Figure 2a). The 0.3 M NaCl elution peak, which exhibited a relatively small peak area and low homogeneity, was not further investigated due to its potential to complicate purification and enrichment processes. CSPsN was obtained by enriching the elution peaks corresponding to distilled water, with a recovery rate of 35.6%. Subsequently, CSPsN was further purified by Sephadex G‐50 gel column chromatography, resulting in the identification of a major peak, designated as CSPsN‐1. CSPsN‐1 is a homogenous polysaccharide with a recovery rate of 58.4%. The protein content of CSPsN‐1, determined by the Bradford method, was 1.20%. The iodine test for starch detection was negative, indicating no presence of starch in CSPsN‐1, thereby conforming to satisfactory purification (Figure S2b).

### 3.3 *M*w determination and monosaccharide composition analysis

**FIGURE 3:**
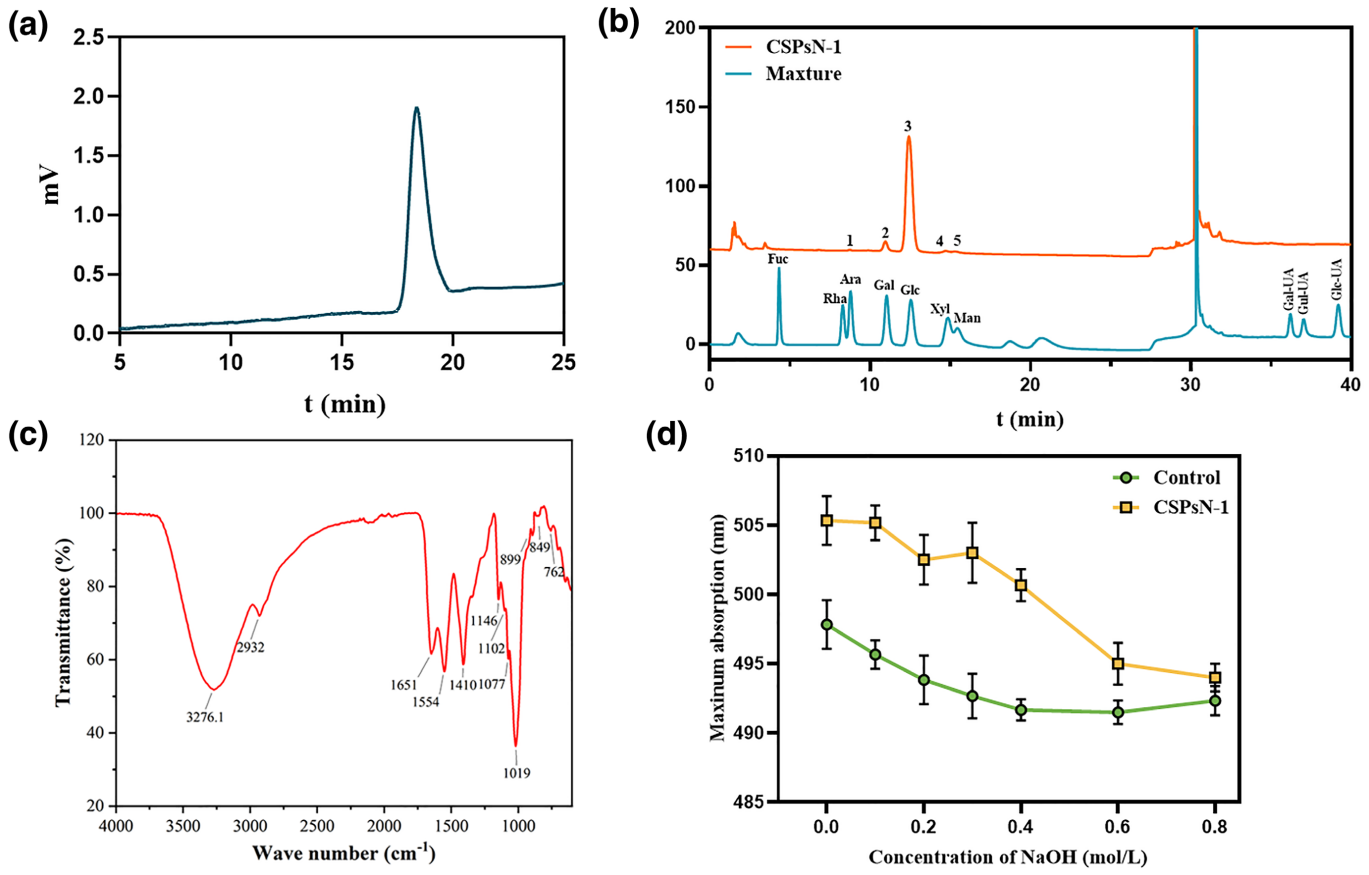
(a) The *M*w curve of CSPsN‐1. (b) Monosaccharide composition maps of CSPsN‐1: 1. Ara, 2. Gal, 3. Glc, 4. Xyl, and 5. Man. (c) Infrared absorption spectra of CSPsN‐1. (d) Triple‐helical conformation analysis of CSPsN‐1.

The HEGPC chromatogram, as depicted in Figure 3a, showed a single symmetrical peak, indicating that CSPsN‐1 has an average *M*w of 7.75 kDa. Figure 3b illustrates the monosaccharide composition analysis of CSPsN‐1, revealing that it comprises arabinose (Ara), galactose (Gal), glucose (Glc), xylose (Xyl), and mannose (Man) in a molar ratio of 0.48: 7.92: 86.39: 2.42: 2.79. This composition indicates that Glc is the predominant monosaccharide component of CSPsN‐1. These findings suggest that the main chain of CSPsN‐1 consists primarily of Glc residues.

### 3.4 Infrared spectra analysis

FT‐IR spectroscopy is an effective technique for the preliminary qualitative assessment of polysaccharide structure by analyzing absorption peaks at specific wavenumbers (cm^−1^). The functional group analysis is typically conducted within the absorption peak range of 4000–1000 cm^−1^, while more detailed structural information can be obtained from the fingerprint region below 1000 cm^−1^.

In the FT‐IR spectra of CSPsN‐1 (Figure 3c), a broad absorption peak spanning from 3500 to 3000 cm^−1^ is observed, attributed to the stretching vibrations of –OH groups. Additionally, a faint peak around 2932 cm^−1^ corresponds to C–H stretching vibrations (Zhang et al., 2021). A significant absorption peak at approximately 1651 cm^−1^ may originate from the stretching of crystal water or C–O (Tian et al., 2023). The peak at 1410 cm^−1^ is likely due to the deformation vibration of C–H bonds (Zhou et al., 2023). Furthermore, strong absorption peaks at 1146, 1077, and 1019 cm^−1^ are attributed to the C–O–C and C–O–H stretching vibrations of glycosidic bonds (Gong et al., 2022). Distinct absorptions at 849 and 899 cm^−1^ indicate the presence of α‐ and β‐glycosidic linkages between the sugar units. A weak absorption peak at 762 cm^−1^ is caused by the symmetric stretching vibration of pyran, further confirming the pyranose structure of CSPsN‐1 (Shi et al., 2023).

### 3.5 Morphological properties

Congo Red forms a complex with polysaccharides that exhibit a triple‐helix structure, leading to a red shift in the maximum absorption wavelength compared to Congo Red alone (Zhang et al., 2021). Figure 3d illustrates the maximum absorption wavelength of the CSPsN‐1 complex. With increasing NaOH concentrations, the maximum absorption wavelength of the Congo Red complex decreases. The absence of a red shift at higher NaOH concentrations suggests that CSPsN‐1 has a nontrihelix structure (Zhu et al., 2021). This observation could be attributed to the high monosaccharide content in the heteropolysaccharide structure, which interferes with molecular cross‐linking and triple‐helix stability, thereby preventing the formation of stable tripe‐helix conformations in solution (Wu et al., 2022).

### 3.6 Structural analysis of CSPsN‐1

#### 3.6.1 Determination of the type of glycosidic linkage

To elucidate the glycosidic linkages in CSPsN‐1, a methylation analysis was conducted, revealing the presence of 12 types of partially methylated alditol acetates (PMAAs) as detailed in Table 1. The analysis identified CSPsN‐1 primarily consists of Glc residues, with 1,4‐Glc*p* being the most abundant at 49.8%, followed by t‐Glc*p* at 29.0% and 1,3,4‐Glc*p* at 7.2%. Minor linkages included 1,4,6‐Glc*p*, 1,3‐Glc*p*, 1,6‐Glc*p*, and 1,3,4,6‐Glc*p*. Additionally, two Gal residues were identified: t‐Gal*p* and 1,4‐Gal*p*, accounting for 3.0% and 2.1%, respectively. Other residues, such as 1,4‐Xyl*p*, 1,2,4‐Man*p*, and 1,2‐Man*p*, were present in smaller amounts. The absence of detectable Ara residues in methylation analysis may be due to their low abundance and potential losses during the methylation process (Chen, Jiang, et al., 2023). These findings suggest that the main chain of CSPsN‐1 is primarily composed of 1,4‐Glc*p*, 1,3,4‐Glc*p*, and t‐Glc*p*.

**TABLE 1:** GC–MS of alditol acetate derivatives from CSPsN‐1.

Methylated sugars	Type of linkage	Molar ratio %	Mass fragments (m/z)
2,3,4,6‐Me_4_‐Glc*p*	Glc*p*‐(1→	29.0	43, 71, 87, 101, 117, 129, 143, 161, 205
2,3,4,6‐Me_4_‐Gal*p*	Gal*p*‐(1→	3.0	43, 71, 87, 101, 117, 129, 143, 161, 205
2,3‐Me_2_‐Xyl*p*	→4)‐Xyl*p*‐(1→	1.5	43, 71, 87, 99, 101, 117, 129, 161, 189
3,4,6‐Me_3_‐Man*p*	→2)‐Man*p*‐(1→	1.4	43, 87, 129, 161, 189
2,4,6‐Me_3_‐Glc*p*	→3)‐Glc*p*‐(1→	1.0	43, 87, 99, 101, 117, 129, 161, 173, 233
2,3,6‐Me_3_‐Gal*p*	→4)‐Gal*p*‐(1→	2.1	43, 87, 99, 101, 113, 117, 129, 131, 161, 173, 233
2,3,6‐Me_3_‐Glc*p*	→4)‐Glc*p*‐(1→	49.8	43, 87, 99, 101, 113, 117, 129, 131, 161, 173, 233
2,3,4‐Me_3_‐Glc*p*	→6)‐Glc*p*‐(1→	0.7	43, 87, 99, 101, 117, 129, 161, 189, 233
2,6‐Me_2_‐Glc*p*	→3,4)‐Glc*p*‐(1→	7.2	43, 87, 97, 117, 129, 149
3,6‐Me_2_‐Man*p*	→2,4)‐Man*p*‐(1→	1.6	43, 87, 99, 113, 129, 173, 189, 233
2,3‐Me_2_‐Glc*p*	→4,6)‐Glc*p*‐(1→	2.4	43, 71, 85, 87, 99, 101, 117, 27, 159, 161, 201, 261
2‐Me_1_‐Glc*p*	→3,4,6)‐Glc*p*‐(1→	0.5	43, 58, 87, 97, 117, 139

#### 3.6.2 NMR spectra analysis

The molecular structure and conformation of CSPsN‐1 were further elucidated using 1D (^1^H and ^13^C) and 2D spectra (^1^H‐^1^H COSY, HSQC, and HMBC). As shown in Figure S3, hydrogen signals from the glycan rings were observed in the 3–4 ppm region, while terminal protons appeared in the 4.5–5.5 ppm region (Han et al., 2021). Anomeric carbon signals were primarily located between 93 and 102 ppm, and the sugar ring carbon signals were found between 60 and 85 ppm (Figure S4) (Liu et al., 2024). Moreover, the 2D NMR spectra provided crucial information on C–H and H–H correlations, which are essential for deducing linkage patterns and the spatial conformations of individual sugar residues (Yao et al., 2021).

**FIGURE 4:**
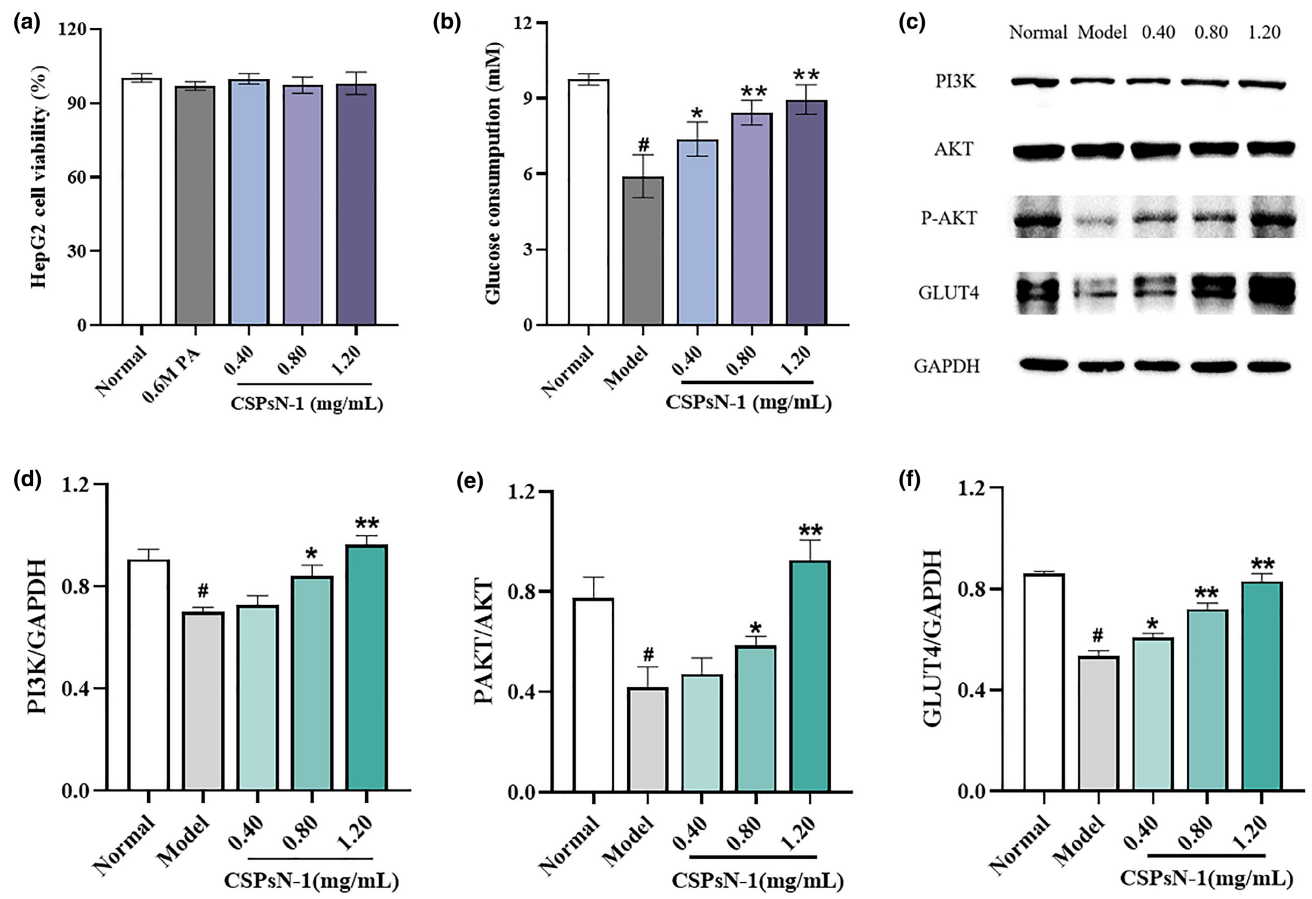
(a) Cell viability of HepG2 cells determined by MTT method. (b) Effect of CSPsN‐1 on the levels of glucose consumption. (c–f) The protein expression levels of PI3K, P‐AKT, and GLUT4. ^#^ *p* < .01 versus the normal control group, *p < .05 versus the PA model group, and **p < 0.01 versus the PA model group.

Attributed to overlapping in 1D NMR and relevant literature, nine cross peaks of anomer protons and carbons were resolved in HSQC spectrum (Figure 4c). These peaks, corresponding to residues A, B, D, E, F, G, I, J, and K, were observed at 5.40/99.55–99.88, 4.64/95.88, 5.06/101.98, 4.66/96.01, 5.23/99.97, 5.07/101.60, 5.39/102.38, 4.96/98.18, and 5.22/92.04, 92.23 ppm, indicating the presence of various glycosidic residues. The detailed NMR data are presented in Table 2.

**TABLE 2:** ^1^H and ^13^C NMR chemical shifts of CSPsN‐1.

Monosaccharide units		Chemical shifts (ppm)					
		C1/H1	C2/H2	C3/H3	C4/H4	C5/H5	C6/H6
A	→4)‐α‐D‐Glc*p*‐(1→	99.55–99.88/5.40	71.60/3.65	73.42/3.93	76.35/3.66	71.41/3.83	60.57/3.86
B	→4)‐β‐D‐Glc*p*‐(1→	95.88/4.64	74.13/3.23	70.08/3.48	75.86/3.83	74.26/3.64	60.42/3.65
D	α‐D‐Glc*p*‐(1→	101.98/5.06	72.98/3.61	74.56/3.94	73.34/3.43	70.10/3.74	60.93/3.83
E	β‐D‐Glc*p*‐(1→	96.01/4.66	73.42/3.25	76.07/3.74	74.68/3.58	73.42/3.61	62.48/3.87
F	→3,4)‐α‐D‐Glc*p*‐(1→	99.97/5.23	74.26/3.56	75.87/3.83	72.78/3.77	73.22/3.90	62.64/3.73
G	α‐D‐Galp‐(1→	101.60/5.07	73.17/3.77	70.18/4.18	69.37/4.05	72.44/3.71	n.d./3.93
I	→4,6)‐α‐D‐Glc*p*‐(1→	102.38/5.39	74.45/3.58	76.05/3.71	78.50/3.68	n.d./3.91	71.64/3.80
J	→3)‐α‐D‐Glc*p*‐(1→	98.18/4.96	74.34/3.57	80.79/3.96	n.d./4.05	n.d.	n.d.
K	α reducing Glc*p*	92.04, 92.23/5.22	69.50/3.55	72.58/3.70	69.78/3.29	n.d.	n.d.

Abbreviation: n.d., not detected.

For residue A, the anomeric signals were obtained at 5.40/99.55–99.88 ppm, with a significant cross peak between the six carbon signals and one hydrogen signal, indicating an α‐linked residue. The downfield shift of C‐4 at 76.35 ppm suggests 1,4 linkages in residue A (Wang et al., 2019). Similarly, residue B displayed an anomeric carbon signal at 95.88 ppm and a corresponding anomeric proton signal at 4.64 ppm, indicating it is a →4)‐β‐D‐Glc*p*‐(1→ residue due to the downfield shift of C‐4 at 75.86 ppm (Gao et al., 2024). Residue D, E, and G exhibited signals at 5.06/101.98, 4.66/96.01, and 5.07/101.60 ppm, respectively, identifying them as terminal Glc*p* and Gal*p* units. The proton signals suggest an α‐configuration for residues D and G, and a β‐configuration for residue E (Bu et al., 2022; Xia et al., 2022; Zhong et al., 2021). The 5.23/99.97 ppm (F H‐1/C‐1) signals, along with downfield peaks of H‐3 (3.83 ppm) and C‐3 (75.87 ppm), and H‐4 (3.77 ppm) and C‐4 (72.78 ppm), were attributed to →3,4)‐α‐D‐Glc*p*‐(1→ (He et al., 2024). Similarly, the peak at 5.39/102.38 ppm was attributed to →4,6)‐α‐D‐Glc*p*‐(1→ (residue I (Ni et al., 2022). Based on the weak cross‐peaks observed in the ^1^H‐^1^H COSY and HSQC spectra, combined with relevant reference data and the methylation results, the chemical shifts at 4.96/98.17 ppm (J H‐1/C‐1) and 4.96/3.57 ppm (J H‐1/H‐2), along with peaks at 3.96 ppm (H‐3) and 80.79 ppm (C‐3), were assigned as →3)‐α‐D‐Glc*p*‐(1→ (Du et al., 2019). For residue K, the C‐1/H‐1 correlation at 92.04, 92.23/5.22 ppm, indicated that residue K was corresponding to free α‐reducing Glc*p*, supported by literature references (Liu et al., 2024; Shi et al., 2019).

Based on the cross peaks observed in the HMBC (Figure 4e), the linkage sequence among the residues of CSPsN‐1 was determined. The signals at 5.40/76.35 ppm (A H‐1/C‐4) and 3.66/99.76 ppm (A H‐4/C‐1) indicated the presence of →4)‐α‐D‐Glc*p*‐(1→4)‐α‐D‐Glc*p*‐(1→ structures. Additional cross peaks at 5.40/72.78 ppm (A H‐1/F C‐4), 5.06/72.78 ppm (D H‐1/F C‐4), and 3.66/101.98 ppm (A H‐4/D C‐1) suggest the following linkages: the O‐1 of residue A is linked to the C‐4 of residue F, the O‐1 of residue D is linked to the C‐4 of residue F, and O‐4 of residue A is linked to the C‐1 of residue D. Due to the low abundance of certain residues and overlapping magnetic signals, the presence or linkage structures of other residues were not detected in NMR spectra. The complete 2D NMR spectrums are shown in Figures S5–S7.

Therefore, the proposed structure of CSPsN‐1 is inferred to be a neutral polysaccharide have a backbone composed of →4)‐α‐D‐Glc*p*‐(1→ and →3,4)‐α‐D‐Glc*p*‐(1→ units, and terminal residues of α‐D‐Glc*p*. This study provides novel structural information on neutral polysaccharides from coix seeds.

### 3.7 Effect of CSPsN‐1 on glucose consumption of cell model

Insulin resistance is characterized by a diminished sensitivity to insulin, resulting in an inability to regulate glucose utilization in tissues, leading to decreased glucose utilization efficiency. This resistance is particularly evident in the liver, where it manifests as reduced glucose consumption. Palmitic acid (PA), a type of free fatty acid, has been demonstrated to directly impair insulin signaling pathways in hepatic cells and pancreatic beta cells (Wang et al., 2023; Zhao, Wu, et al., 2016).

Insulin resistance was induced in HepG2 cells (IR‐HepG2) by treating them with 0.6 mM PA for 24 h. As shown in Figure 4b, there was a significant reduction (*p* < .05) in glucose uptake in the model group, indicating the successful establishment of the IR‐HepG2 cell model. Glucose consumption in IR‐HepG2 cells increased in a dose‐dependent manner following treatment with different concentrations of CSPsN‐1 (0.40, 0.80, and 1.20 mg/mL). Specifically, glucose consumption increased to 8.43 mM and 8.95 mM at CSPsN‐1 concentrations of 0.80 and 1.20 mg/mL, respectively, which were approximately 1.43 and 1.51 times higher than that of the model group. Furthermore, the MTT assay indicated that varying concentrations of CSPsN‐1 had minimal impact on cytotoxicity (Figure 4a). These findings suggest that CSPsN‐1 can enhance glucose consumption in IR‐HepG2 cells.

### 3.8 Effect of CSPsN‐1 on the insulin signaling pathways

Dysregulation of glucose and lipid metabolism disrupts insulin signal transduction and reduces insulin sensitivity. Improving insulin resistance depends on activating the cellular insulin pathway. Insulin regulates this pathway by binding to its receptor, which activates the insulin receptor substrate (IRS). The activated IRS then binds to and activates PI3K, initiating the PI3K/AKT signaling pathway. This activation enhances GLUT4 translocation from the intramembrane region to the plasma membrane, thereby promoting increased glucose uptake and utilization (Gong et al., 2017; Jiang et al., 2023).

To evaluate the impact of CSPsN‐1 on the PI3K/AKT signaling pathway in IR‐HepG2 cells, we measured the expression levels of key proteins involved in glucose transport: PI3K, total AKT, P‐AKT, and GLUT4. As shown in Figure 4c–f, the model group exhibited decreased expression of PI3K, P‐AKT, and GLUT4. However, treatment with CSPsN‐1 promoted the expression of these proteins in a dosage‐dependent manner. These results suggest that CSPsN‐1 improves glucose metabolism and insulin resistance via activation of the PI3K/AKT/GLUT4 signaling pathway.

### 3.9 Discussion

Recent research has highlighted the significant biological activities of natural polysaccharides, especially their antidiabetic effects through improving insulin resistance and promoting glucose metabolism (Lai et al., 2024; Zhu et al., 2024). Although studies on coix seed polysaccharides are limited, existing evidence indicates their promising bioactivity. This study aimed to optimize the extraction process for coix seed polysaccharides, analyze the structure of homogeneous polysaccharides, and investigate their activity on insulin resistance, laying the groundwork for future research on their mechanisms of action and potential therapeutic applications in metabolic disorders.

Polysaccharide extraction is influenced by various factors, and different combinations can affect the yield and properties of the active components. Determining the optimal combination is crucial to maximize yield and conserve energy. The Box–Behnken design model was used to optimize the extraction process by establishing quantitative relationships between variables, ranking their importance and interactions, predicting outcomes, and identifying the optimal process (Lin et al., 2023). Under these optimized conditions, the yield of coix seed polysaccharide was enhanced, resulting in the isolation of a novel neutral polysaccharide, CSPsN‐1.

Polysaccharides exhibit significant bioactivities that are closely associated with their molecular weight, monosaccharide composition, and glycosidic linkages. Research on the relationship between the structural composition and biological activity of polysaccharides from coix seed remains limited, but some scientific inferences can be drawn from existing literature. Previous investigations have indicated that the *M*w of polysaccharides exhibiting hypoglycemic effects typically ranges from 3.0 to 150.0 kDa (Zhang & Li, 2018). For example, polysaccharides with an average *M*w of 5.28 kDa have demonstrated favorable hyperglycemic activity (Chen et al., 2020). Similarly, fucoidan with a low molecular weight (5–30 kDa) has been shown to significantly reduce blood glucose levels (Kim et al., 2012). In this study, CSPsN‐1 has a molecular weight comparable to those reported in above inference and has likewise shown good hypoglycemic activity. Studies have demonstrated that polysaccharides composed of Gal, Ara, and Glc can significantly enhance insulin resistance and glucose intolerance, with high Glc content being responsible for their hypoglycemic activities (Wang et al., 2016). Therefore, the hypoglycemic activity of CSPN‐1 could be attributed to its substantial concentration of Glc. In addition, it has been reported that some α‐Glc*p* residues can act as bioactive macromolecules to help control hyperglycemia. For instance, Zhang et al. (2019) isolated α‐Glc*p* from *Euryale ferox* Salisb. seeds, which increased glucose consumption in insulin‐resistant HepG2 and 3 T3‐L1 cells. The neutral polysaccharide from sea cucumber *Stichopus japonicus*, mainly composed of →4)‐α‐D‐Glc*p*‐(1→, has a significant effect on improving insulin resistance via the AKT/GSK3β signaling pathway (Gong et al., 2022). Similarly, β‐D‐Glc*p* and polysaccharides with 1→4 β‐glycosidic linkages extracted from cereals, fungi, and other natural sources have demonstrated antidiabetic activities (Wan et al., 2021). Therefore, the regulating hyperglycemia effect of CSPsN‐1 may be positively related to its specific forms of glycosidic linkages, including →4)‐α/β‐D‐Glc*p*‐(1→, →3,4)‐α‐D‐Glc*p*‐(1→, and α/β‐D‐Glc*p*‐(1→.

The liver is a primary target organ for insulin and plays a crucial role in energy and glucose metabolism. In insulin resistance, the insulin signaling pathway is impaired, leading to metabolic dysfunctions (Xue et al., 2023). CSPsN‐1 enhances glucose uptake in IR‐HepG2 cells by activating PI3K, increasing AKT (Ser473) phosphorylation, and normalizing GLUT4 levels. These findings suggest that CSPsN‐1 improves insulin resistance in HepG2 cells by modulating the PI3K/AKT/GLUT4 pathway.

As a low‐molecular‐weight glucan, CSPsN‐1 can effectively penetrate tissue barriers to reach cells and exert its biological activity (Tian et al., 2023). Its high content of 1,4‐glycosidic bonds makes it an effective dietary fiber, helping to slow blood glucose rise and improve insulin sensitivity. Despite its promising benefits, the structure–activity relationship underlying its hypoglycemic effect is not fully understood due to its polysaccharide complexity. In vitro studies indicate that coix seed, a nutritious grain, has beneficial effects in managing diabetes and associated risks. Further comprehensive in structure–activity relationship and vivo studies are necessary to fully understand the mechanisms and to design polysaccharides with targeted biological functions.

## 4 CONCLUSIONS

In this research, an ultrasonic‐assisted enzymatic extraction technique was employed to extract polysaccharides from coix seeds. Key extraction parameters were optimized using single‐factor experiments and RSM, significantly enhancing the extraction efficiency. The combined action of ultrasound and complex enzyme accelerated dissolution and induced structural modifications in the extracted polysaccharides, thereby influencing their higher‐order structures and biological activities. A novel neutral polysaccharide (CSPsN‐1) was isolated and purified from coix seed. The preliminary structure of CSPsN‐1 was characterized using *M*w determination, monosaccharide composition analysis, FT‐IR spectroscopy, Congo Red test, methylation analysis, and NMR spectroscopy. CSPsN‐1 was found to have a *M*w of 7.75 kDa. Its backbone chain consists of →4)‐α‐D‐Glc*p*‐(1→, →3,4)‐α‐D‐Glc*p*‐(1→ units, with terminal residues of α‐D‐Glc*p*. Bioactivity evaluations indicated that CSPsN‐1 significantly enhances glucose consumption and ameliorates PA‐induced insulin resistance in HepG2 cells by activating the PI3K/AKT/GLUT4 signaling pathway. The hypoglycemic potential of CSPsN‐1 is likely attributed to its specific polysaccharide characteristics. The high molecular weight and diverse structural forms of D‐Glc*p* within CSPsN‐1 can reduce glucose production and absorption while enhancing insulin sensitivity, making it beneficial for diabetes therapy. These results may improve the use of coix seed as functional ingredient with hypoglycemic effects. The detailed correlations between the structural properties and bioactivities of coix seed polysaccharides will be further studied in our later work.

## AUTHOR CONTRIBUTIONS

**Guozhen Wu:** Data curation (equal); investigation (equal); methodology (equal); writing – original draft (equal). **Shuang Liu:** Conceptualization (equal); methodology (equal); supervision (equal). **Zhenqiang Wang:** Investigation (equal); methodology (equal); resources (equal). **Xiao Wang:** Project administration (equal); supervision (equal); writing – review and editing (equal).

## CONFLICT OF INTEREST STATEMENT

The authors declare no conflicts of interest.

## Supporting information


Appendix S1.

## Data Availability

The data that support the findings of this study are available from the corresponding author upon reasonable request.
