# Supplementary material for: Structural characteristics of neutral polysaccharides purified from coix seed and its anti‐insulin resistance effects on HepG2 cells
Source: Food Sci Nutr. 2024 Sep 8;12(10):8419–31. doi: 10.1002/fsn3.4402 (PMC11521644; doi:10.1002/fsn3.4402)
Supplement: Supplementary file 1 — Appendix S1. [file FSN3-12-8419-s001.docx]

**Supplementary data**

**FIGURE S1** Effect of various factors on the yield of crude polysaccharides from coix seed.


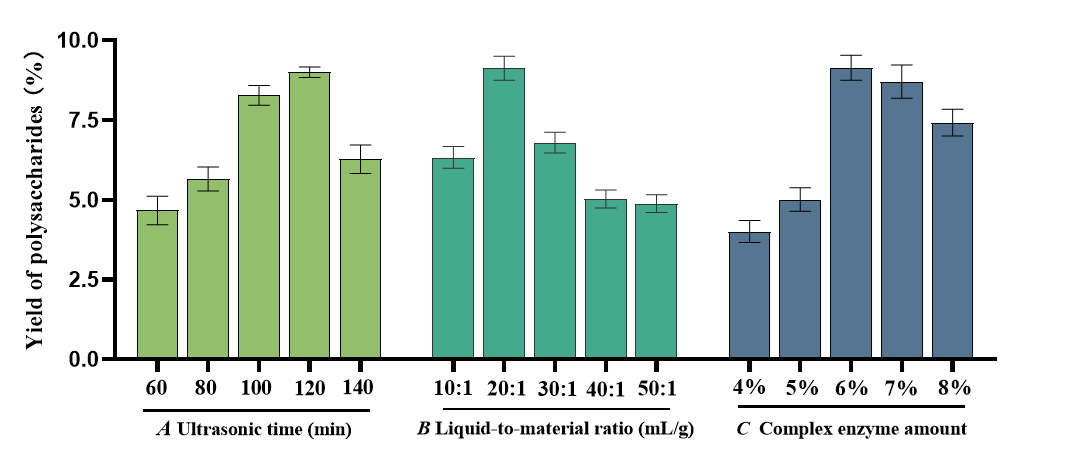


**FIGURE S2** (A) Standard curve of BSA, (B) Inodine test: a. Starch solution (1mg/mL), b. CSPsN-1 solution (5 mg/mL).


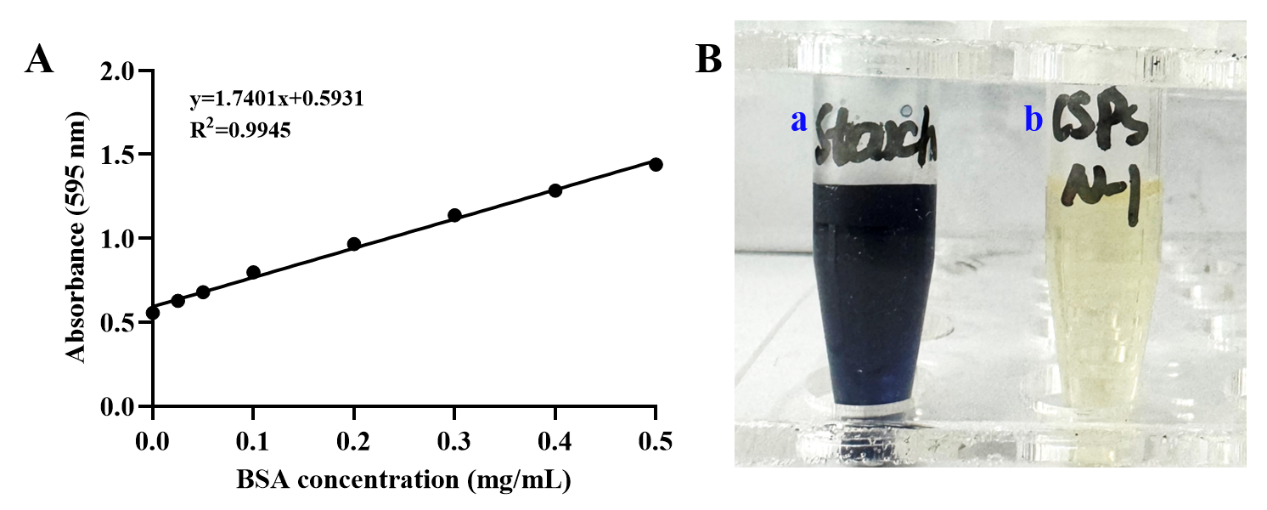


**FIGURE S3** ^1^H NMR spectrum


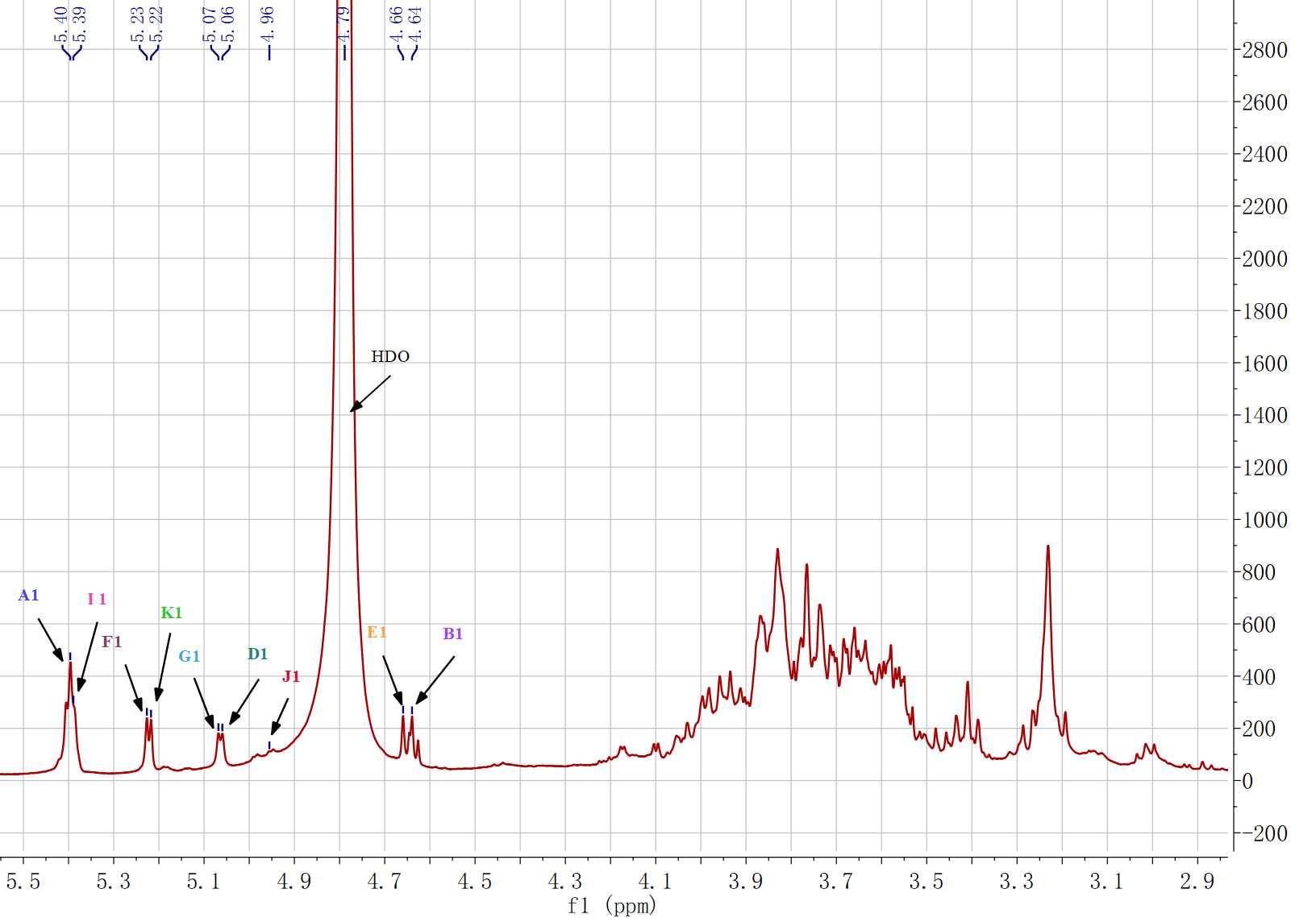


**FIGURE S4** ^13^C NMR spectrum


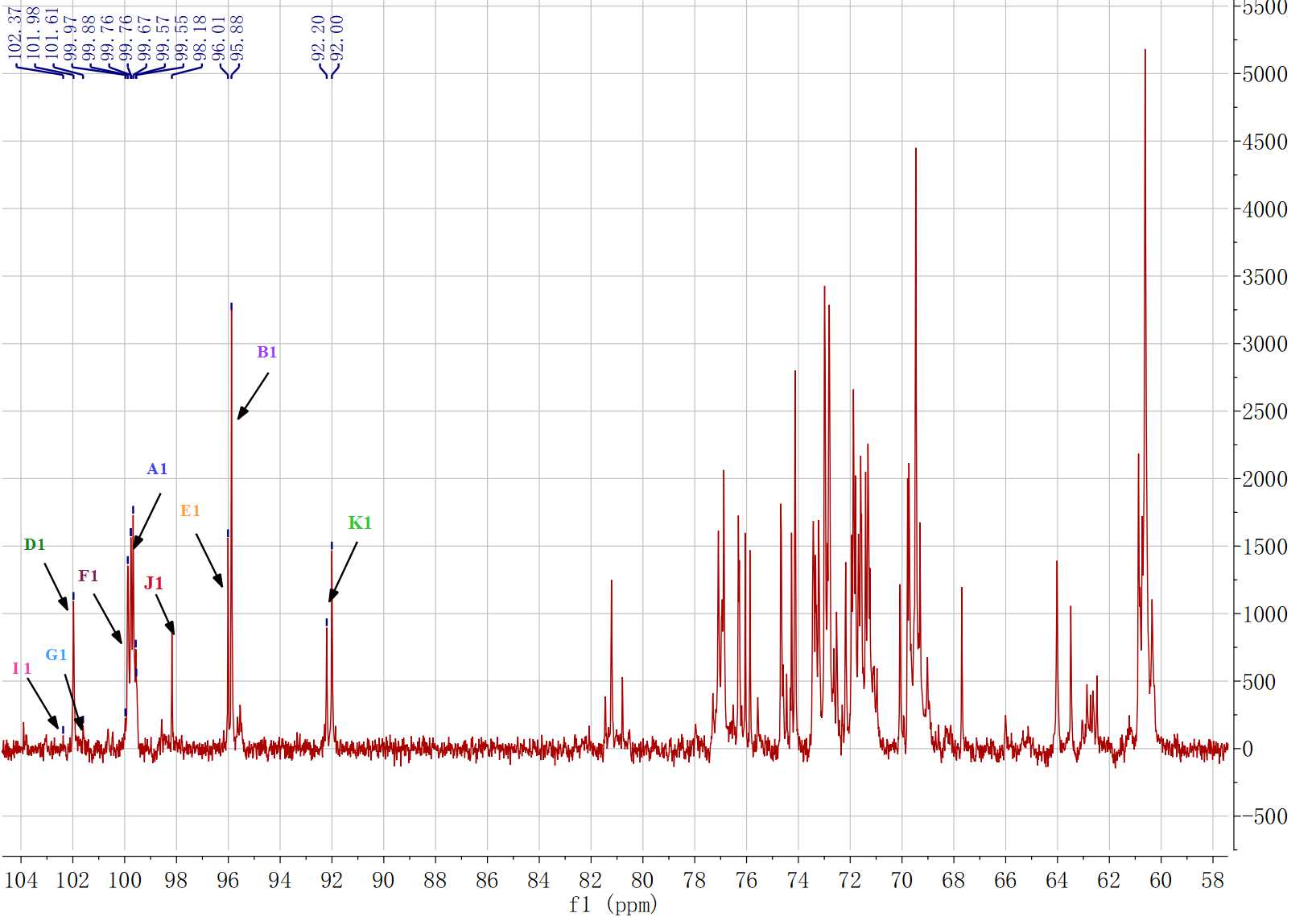


**FIGURE S5** ^1^H-^1^H COSY spectrum


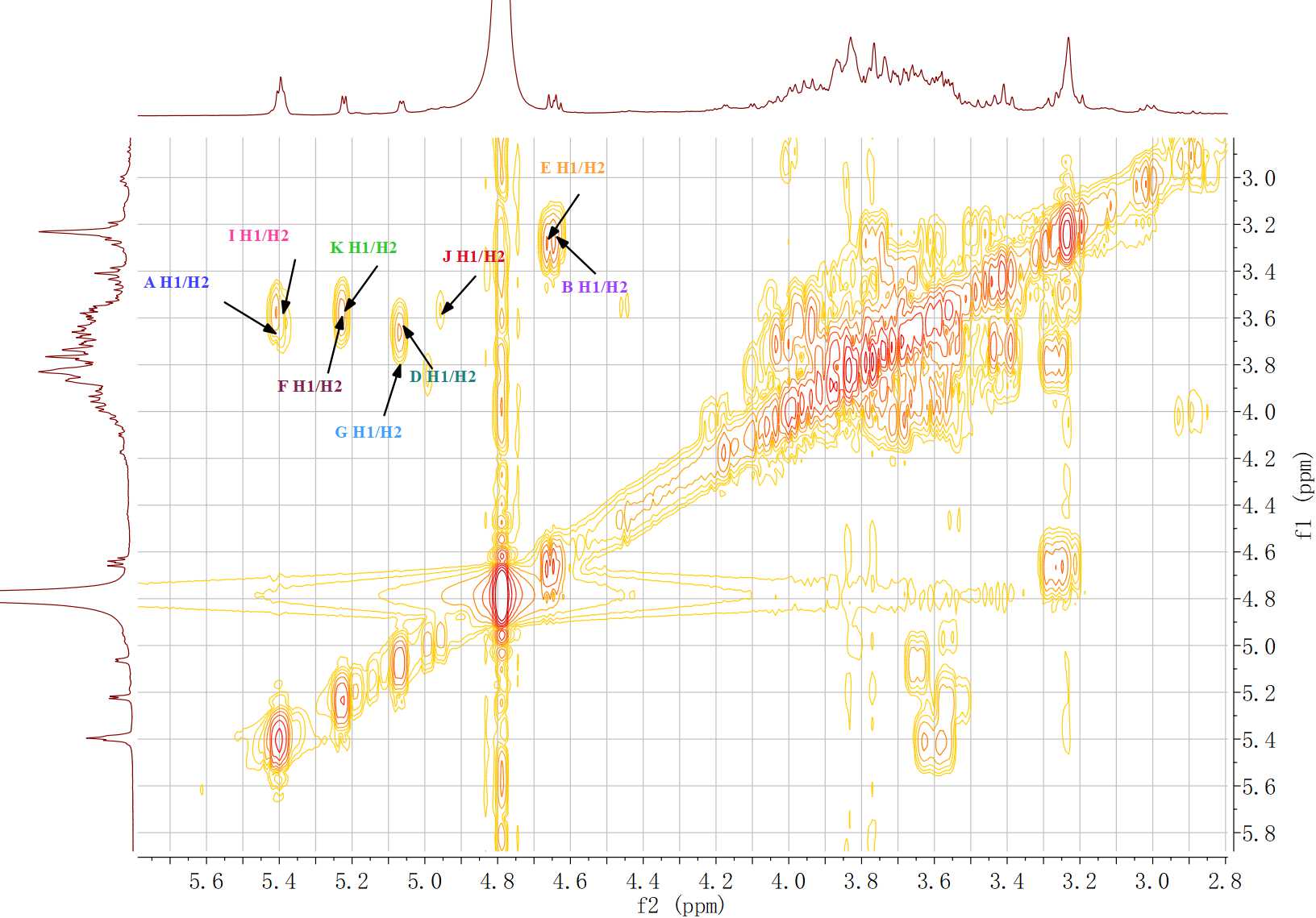


**FIGURE S6** HSQC spectrum


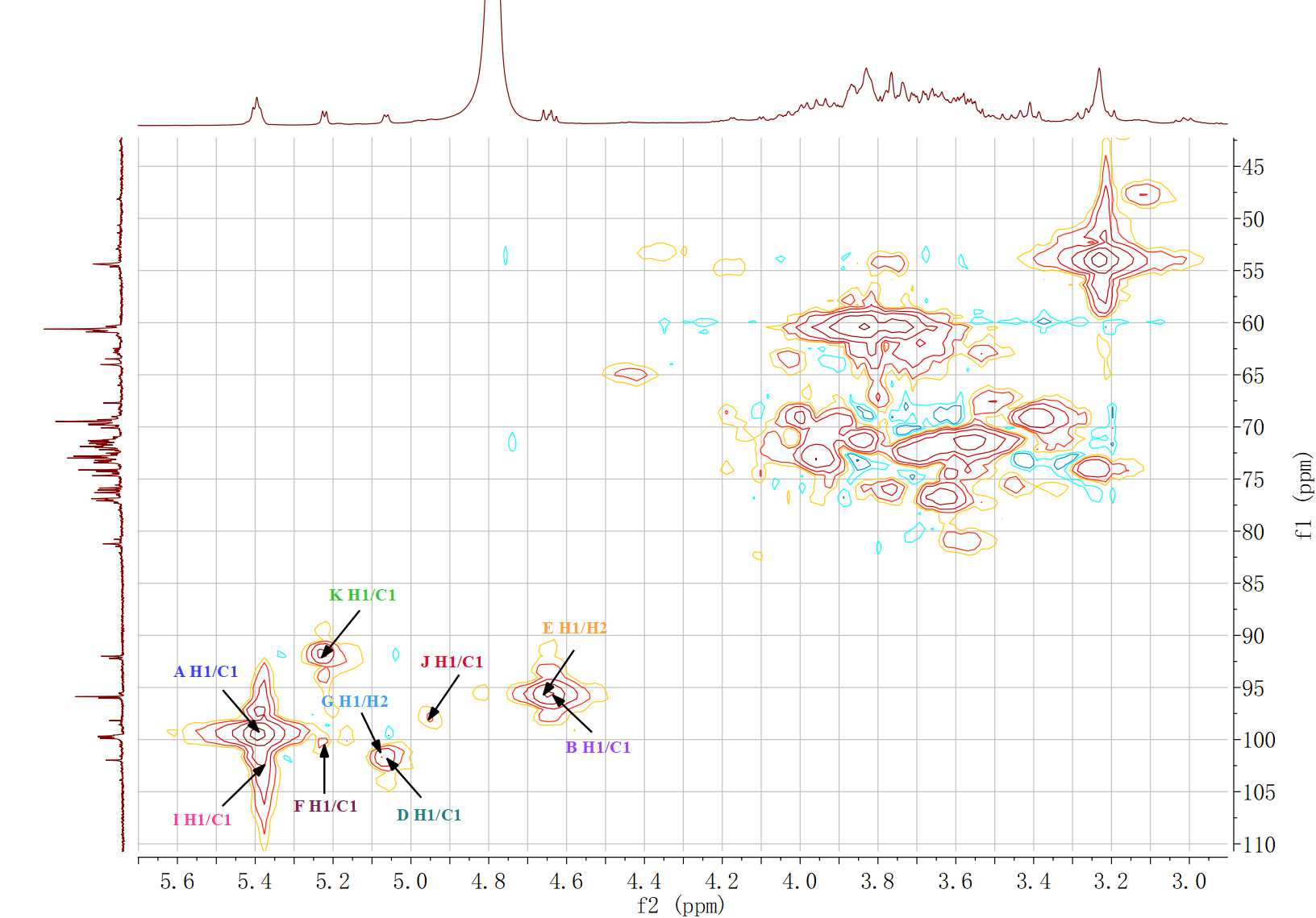


**FIGURE S7** HMBC spectrum


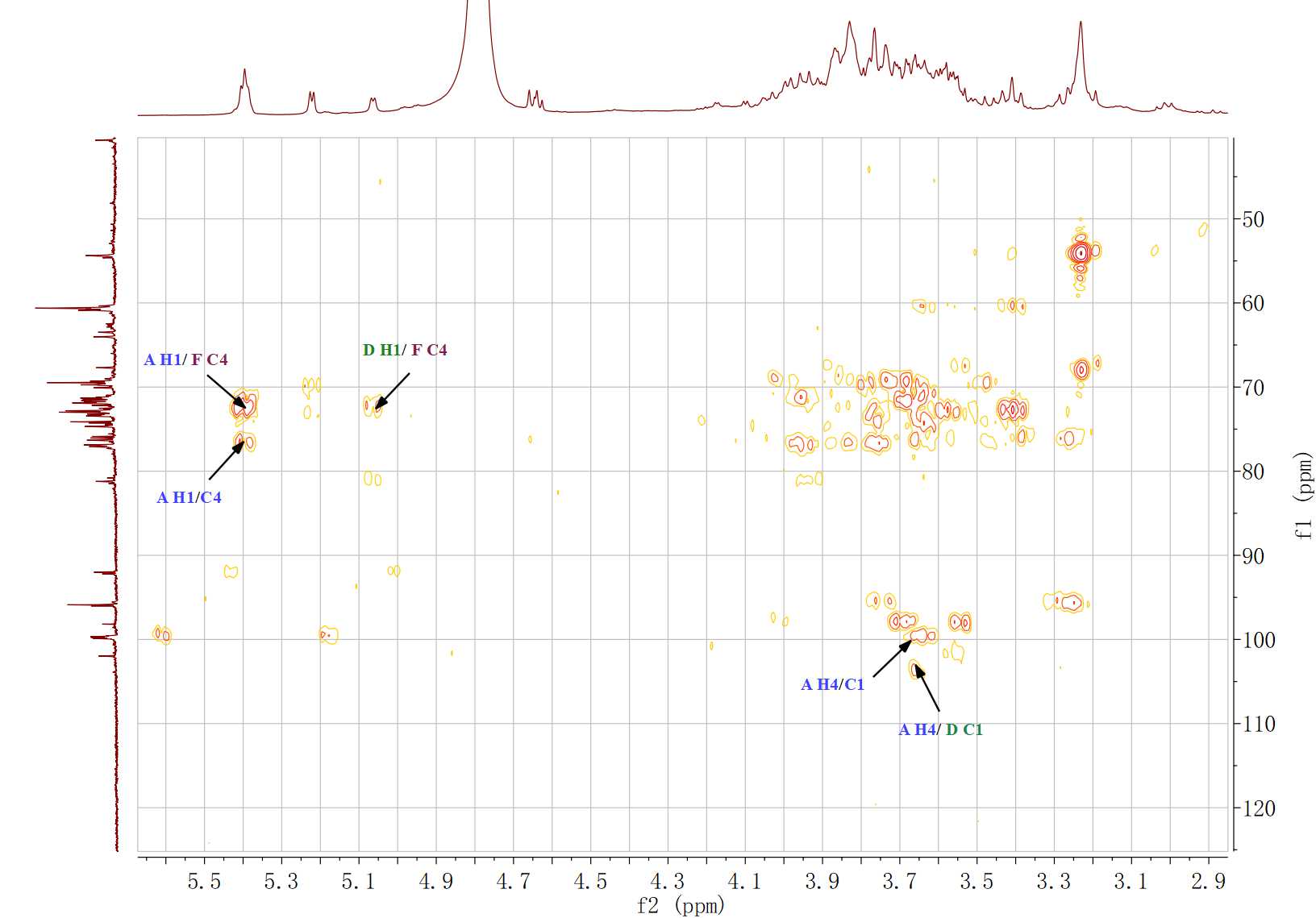


**TABLE S1** Box-Behnken design and results for the yield of the crude polysaccharides from coix seed.

| Run order | Independent variable | | |  | Investigated response |
| --- | --- | --- | --- | --- | --- |
|  | Variable *A*: Ultrasonic time (min) | Variable *B*: Liquid-to-material ratio (mL/g) | Variable *C*: Complex enzyme amount (%) |  | Yield of polysaccharides (%) |
| 1 | 120 | 20:1 | 6 |  | 9.38 |
| 2 | 100 | 30:1 | 6 |  | 5.38 |
| 3 | 100 | 20:1 | 5 |  | 5.49 |
| 4 | 140 | 20:1 | 5 |  | 7.74 |
| 5 | 140 | 30:1 | 6 |  | 7.45 |
| 6 | 120 | 30:1 | 5 |  | 5.64 |
| 7 | 120 | 20:1 | 6 |  | 9.02 |
| 8 | 120 | 30:1 | 7 |  | 6.76 |
| 9 | 120 | 20:1 | 6 |  | 9.66 |
| 10 | 140 | 20:1 | 7 |  | 8.22 |
| 11 | 140 | 10:1 | 6 |  | 4.60 |
| 12 | 120 | 10:1 | 7 |  | 5.79 |
| 13 | 120 | 20:1 | 6 |  | 9.59 |
| 14 | 120 | 20:1 | 6 |  | 9.32 |
| 15 | 100 | 20:1 | 7 |  | 6.85 |
| 16 | 120 | 10:1 | 5 |  | 4.51 |
| 17 | 100 | 10:1 | 6 |  | 4.49 |

**TABLE S2** Analysis of variance (AVONE) for the fitted quadratic polynomial model of polysaccharides extraction.

| Source | Sun of square | | DF | | *F*-value | *P*-value | | | Significance |
| --- | --- | --- | --- | --- | --- | --- | --- | --- | --- |
| Model | 5774.80 | | 9 | | 44.53 | ＜0.0001 | | | *** |
| *A* | 420.13 | | 1 | | 29.15 | 0.001 | | | ** |
| *B* | 426.87 | | 1 | | 29.62 | 0.001 | | | ** |
| *C* | 224.78 | | 1 | | 15.60 | 0.0055 | | | ** |
| *AB* | 95.69 | | 1 | | 6.64 | 0.0366 | | | * |
| *AC* | 19.71 | | 1 | | 1.37 | 0.2805 | | |  |
| *BC* | 0.65 | | 1 | | 0.045 | 0.8380 | | |  |
| *A*^2^ | 686.36 | | 1 | | 47.63 | 0.0002 | | | ** |
| *B*^2^ | 3018.55 | | 1 | | 209.47 | ＜0.0001 | | | *** |
| *C*^2^ | 494.77 | | 1 | | 34.33 | 0.0006 | | | ** |
| Residual | 100.87 | | 7 | |  |  | | |  |
| Lack of fit | 64.16 | | 3 | | 2.33 | 0.2158 | | | Not significant |
| Pure error | 36.72 | | 4 | |  |  | | |  |
| R^2^ = 0.9828 | | R^2^_adj_ = 0.9608 | | *C*.*V*.(%) = 5.37 | | |  |  |  |

*Significant value at < 0.05; ** < 0.01; *** < 0.001 level
